# Toxoplasmic Retinochoroiditis: Clinical Characteristics and Visual Outcome in a Prospective Study

**DOI:** 10.1371/journal.pntd.0004685

**Published:** 2016-05-02

**Authors:** Ana Luisa Quintella do Couto Aleixo, André Luiz Land Curi, Eliezer Israel Benchimol, Maria Regina Reis Amendoeira

**Affiliations:** 1 Infectious Ophthalmology Laboratory, Evandro Chagas National Institute of Infectious Diseases–FIOCRUZ, Rio de Janeiro, Brazil; 2 Toxoplasmosis Laboratory, Oswaldo Cruz Institute–FIOCRUZ, Rio de Janeiro, Brazil; University of Cambridge, UNITED KINGDOM

## Abstract

**Purpose:**

To ascertain the clinical features and visual outcome of toxoplasma retinochoroiditis in a large series of cases.

**Subjects and Methods:**

Two hundred and thirty subjects diagnosed with active toxoplasma retinochoroiditis were prospectively followed for periods ranging from 269 to 1976 days. All patients presented with active retinochoroiditis and positive IgG *T*. *gondii* serology at the beginning of the study and received a standardized drug treatment for toxoplasmosis, both in the first episode and in the subsequent recurrences.

**Results:**

The group involved 118 (51.3%) men and 112 (48.7%) women, with ages ranging from 14 to 77 years, mean of 32.4 years (SD = 11.38). Primary retinochoroidal lesions were observed in 52 (22.6%) cases and active retinochoroiditis combined with old scars in 178 (77.4%) subjects at the beginning of the study. A hundred sixty-two recurrent episodes in 104 (45.2%) patients were observed during follow-up. New subclinical retinochoroidal lesions were detected in 23 of 162 (14.2%) recurrences episodes during the follow-up. Posterior segment complications were observed in 73 (31.7%) subjects. Retinochoroidal lesions adjacent to the optic nerve and in the macular area were observed in 27 of 40 (67.5%) cases of severe visual impairment (VA = 20/200 or worse).

**Conclusion:**

Toxoplasma retinochoroiditis in this population had a high recurrence rate after an active episode. Severe visual impairment was associated with location of the retinochoroidal scar, recurrences and posterior segment complications. It is crucial to consider the location of the lesion in studies analyzing visual prognosis as a measure for treatment effectiveness and prevention strategies.

## Author Summary

Ocular toxoplasmosis affects millions of people worldwide and is a cause of severe vision loss. Prospective studies on the disease are rare and require long and expensive follow-ups. A network of intricate host and parasite factors can influence its evolution and its treatment is still subject of discussion and controversy. This article describes clinical features in a series of cases prospectively followed, confirming findings of previous studies conducted in other continents, despite the probable genetic variability of the evaluated populations and parasite. In addition, brings insights into the course of the disease and causes of visual impairment that may help future studies on strategies for treatment and prevention.

## Introduction

Ocular involvement by *Toxoplasma gondii* may cause severe visual impairment and is the main etiology of posterior uveitis in many parts of the world, however questions about the course of the disease remain unanswered.[1–3] Up to this day there is no consensus on treatment effectiveness and risk factors for the occurrence and recurrence of this eye disease. [2,4–6] Brazil hosts the highest prevalence ever described for ocular toxoplasmosis with a preponderance of different strains from those that predominate in Europe and North America, this alone would possibly justify clinical differences and long term outcome.[7–10] The study of ocular toxoplasmosis offers many challenges to researchers, mainly because of the plethora of factors that may affect the course of the disease and the need of long term follow-ups.

To observe outcomes of toxoplasma retinochoroiditis (TRC) and especially the causes of vision loss in affected patients it is crucial to guide the analysis of prevention strategies and treatment. The study herein presented describes the clinical characteristics of a series of prospectively followed cases of toxoplasma retinochoroiditis, analyzing the course of the disease, recurrence frequency and factors that may influence the visual prognosis.

## Methods

A prospective observational and non-experimental study with 230 subjects with active TRC consulted at the outpatient unit of the Infectious Ophthalmology Laboratory of Evandro Chagas National Institute of Infectious Diseases (INI) was conducted after approval by the INI Ethics Committee (CAAE 0075.0.009.00011). These individuals were selected by the same ophthalmologist from 1912 patients examined between January 2010 and January 2014 of which 820 (42.9%) were referred as probable TRC. Two hundred seventy four individuals were considered eligible with diagnosis of active toxoplasma retinochoroiditis and no exclusion criteria at admission and agreed to participate in the research signing an informed consent. Subjects between 14 and 18 years old who agreed to participate also signed an informed consent in conjunction with a parent or surrogate. Twenty six subjects (9.5%) missed follow-up consultation and 18 (6.5%) met exclusion criteria during the follow-up and were not included in the analysis. All procedures performed with the subjects were approved by the INI Ethic Committee and followed the standard routine of the outpatient unit for ocular toxoplasmosis.

**Fig 1:**
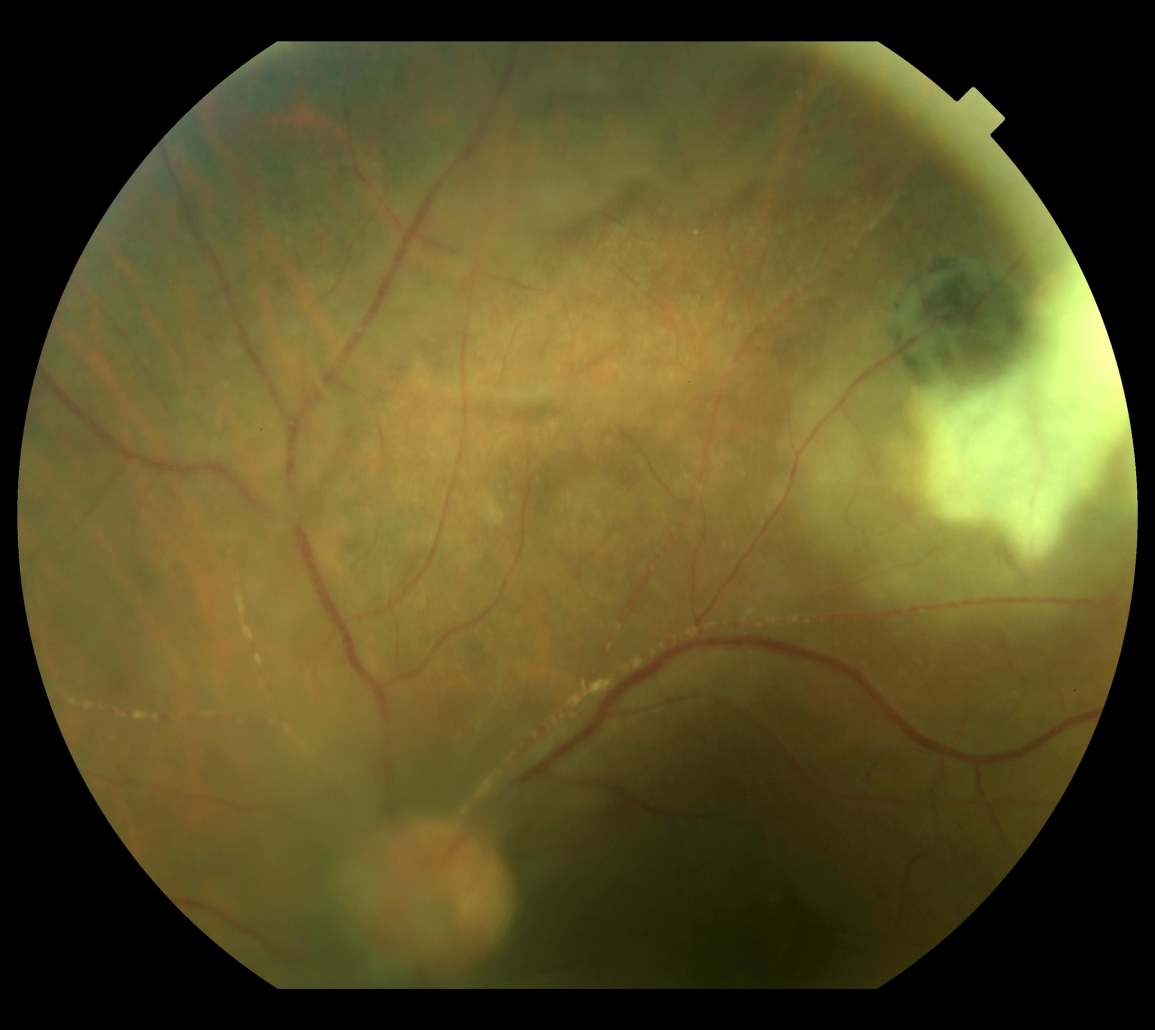
Retinography of a subject with focal exudative retinochoroiditis associated with a pigmented scar. Credits: ALQCA.

**Fig 2:**
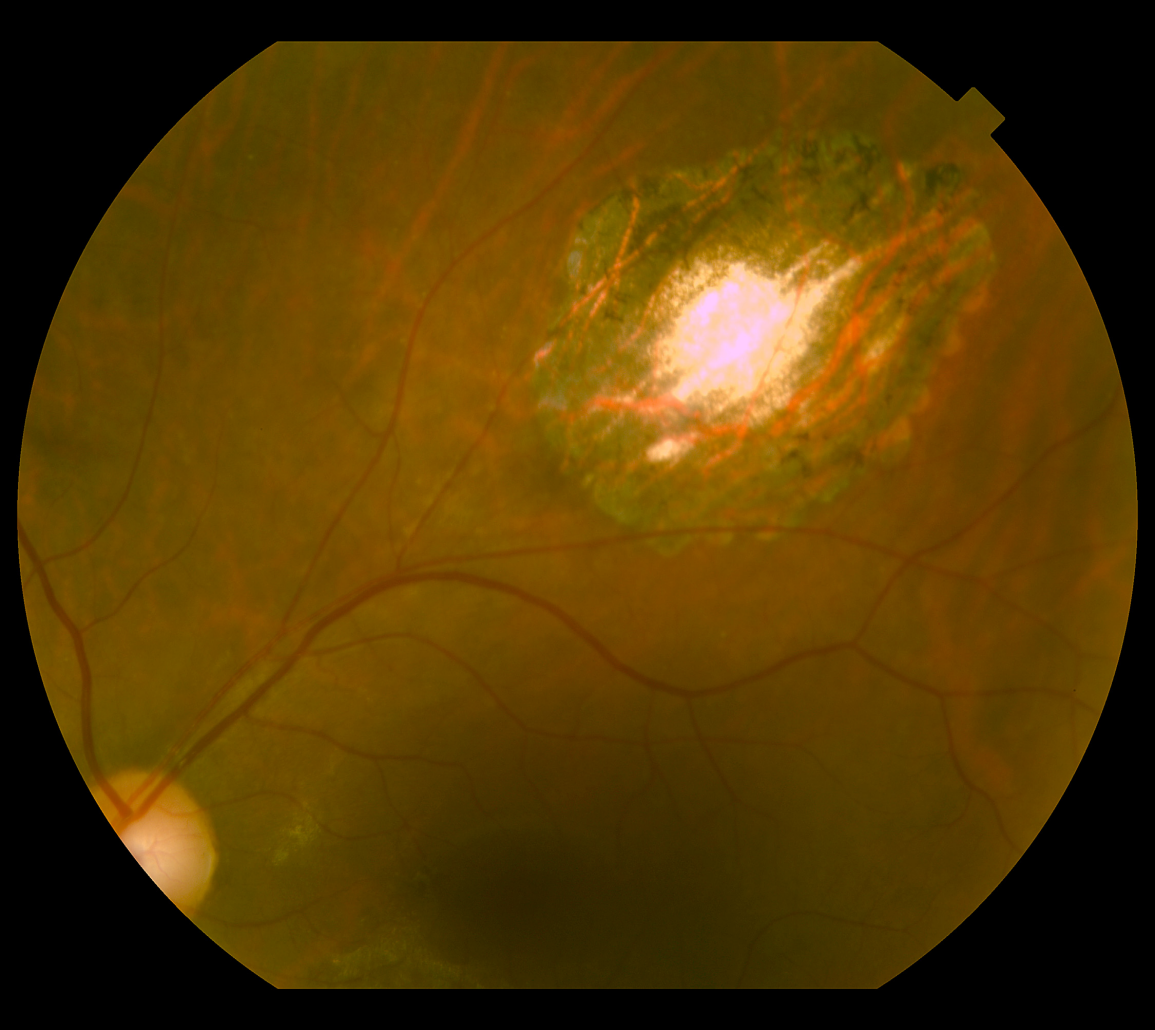
Retinography of the same subject of Fig 1 after standardized drug scheme for toxoplasmosis, showing healed lesions (scars). Credits: ALQCA.

**Fig 3:**
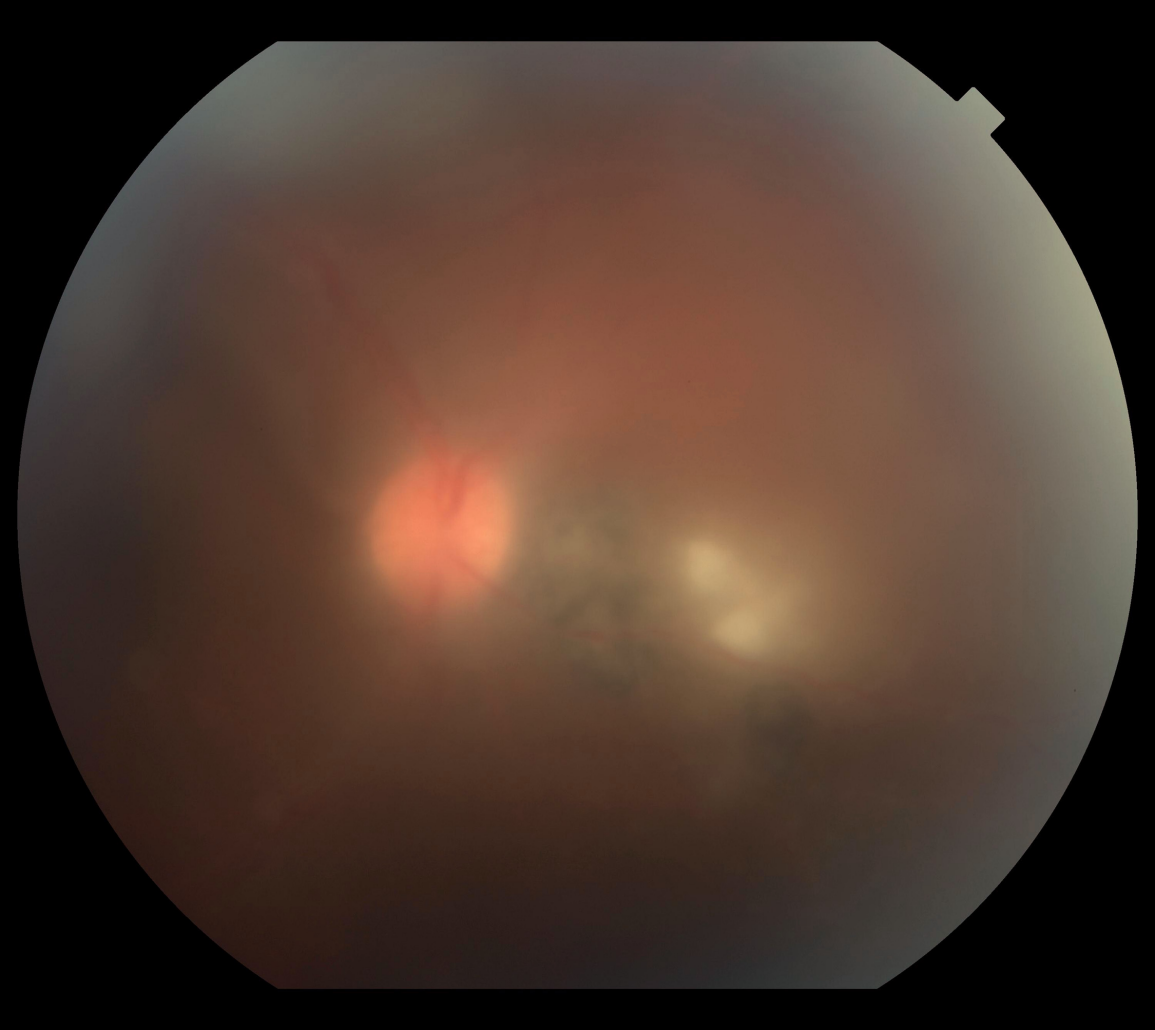
Retinography of a subject with focal exudative retinochoroiditis associated with a pigmented scar. Credits: ALQCA.

**Fig 4:**
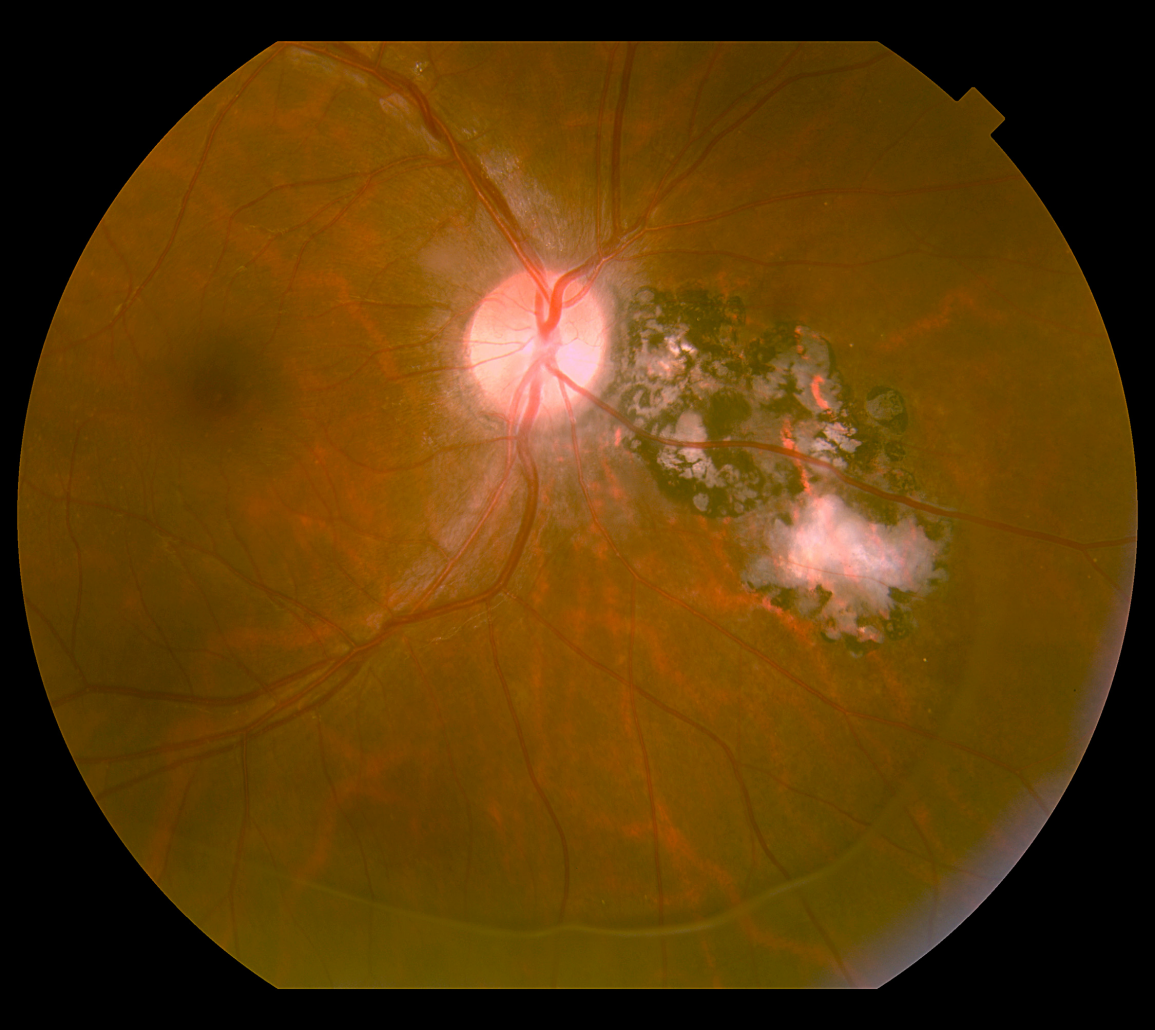
Retinography of the same subject of Fig 2 after standardized drug scheme for toxoplasmosis, showing healed lesions (scars). Credits: ALQCA.

For the purpose of this study, the diagnosis of active toxoplasmic retinochoroiditis was based on clinical criteria formulated by Holland *et al* in patients seropositive for *T*. *gondii* [11] Primary TRC was defined as creamy-white exudative focal retinochoroiditis not associated with retinochoroidal scars in either eye. Recurrent TRC was defined as a focal active retinochoroiditis associated with retinal scarring in the same or contralateral eye (Figs 1–4).[7]

Subclinical episodes were defined as new healed or active lesions observed in fundoscopic examination (by counting the number of lesions described in the admission consultation or comparing retinographies) in subjects who didn’t exhibit new significant symptoms. Significant symptoms were defined as those referred spontaneously by the patient. Episodes of anterior segment inflammation in eyes with retinochoroidal scars had previously been described on patients with ocular toxoplasmosis and were not considered recurrences.[11] Subjects with co-morbidities such as chronic renal failure, systemic infections as AIDS, syphilis and tuberculosis were not considered eligible as well as those with diagnosed auto immune diseases. History of intravenous drugs use, cancer chemotherapy, immunosuppressive drugs or peri and intraocular steroids were considered exclusion criteria. Subjects with single and unilateral exudative retinochoroiditis with positive diagnostic tests for syphilis (VDRL and TPHA, imunoflocculation and hemagglutination for *T*. *pallidum*) and HIV 1 and 2 (rapid test and / or serology by ELISA) performed in the Laboratory of Immunology and Immunogenetics of the INI were also excluded. Individuals with single lesions who did not respond to treatment with the standardized drug scheme for toxoplasmosis during the first 45 days and who were pregnant during any recurrent episodes were also excluded, as well as subjects with multiple exudative lesions of retinochoroiditis.

### Follow-up

Follow-up visits were scheduled within 30 or 45 days and every year after the initial consultation. Yearly returns were programmed for every subject as well as individual consultations according to the presence of complications. Subjects were instructed to seek the Infectious Ophthalmology Laboratory of INI at any time in case of eye symptoms, especially blurred vision, red eye or floaters. Subjects who did not return on the scheduled dates were contacted by phone or mail. A minimal of three consultations was required to consider the patient followed, one for admission, the second within 45 days after the first one and the third between 8 months after the first visit and July 2015. Retinography was performed for assessment of retinal status before and after treatment, whenever the transparency of the ocular media and the patient allowed and depending on the availability of the retinal camera. For the purpose of this study visual acuity (VA) of 20/200 or worse with the best correction in the affected eye was considered severe vision impairment.[12]

**Fig 5:**
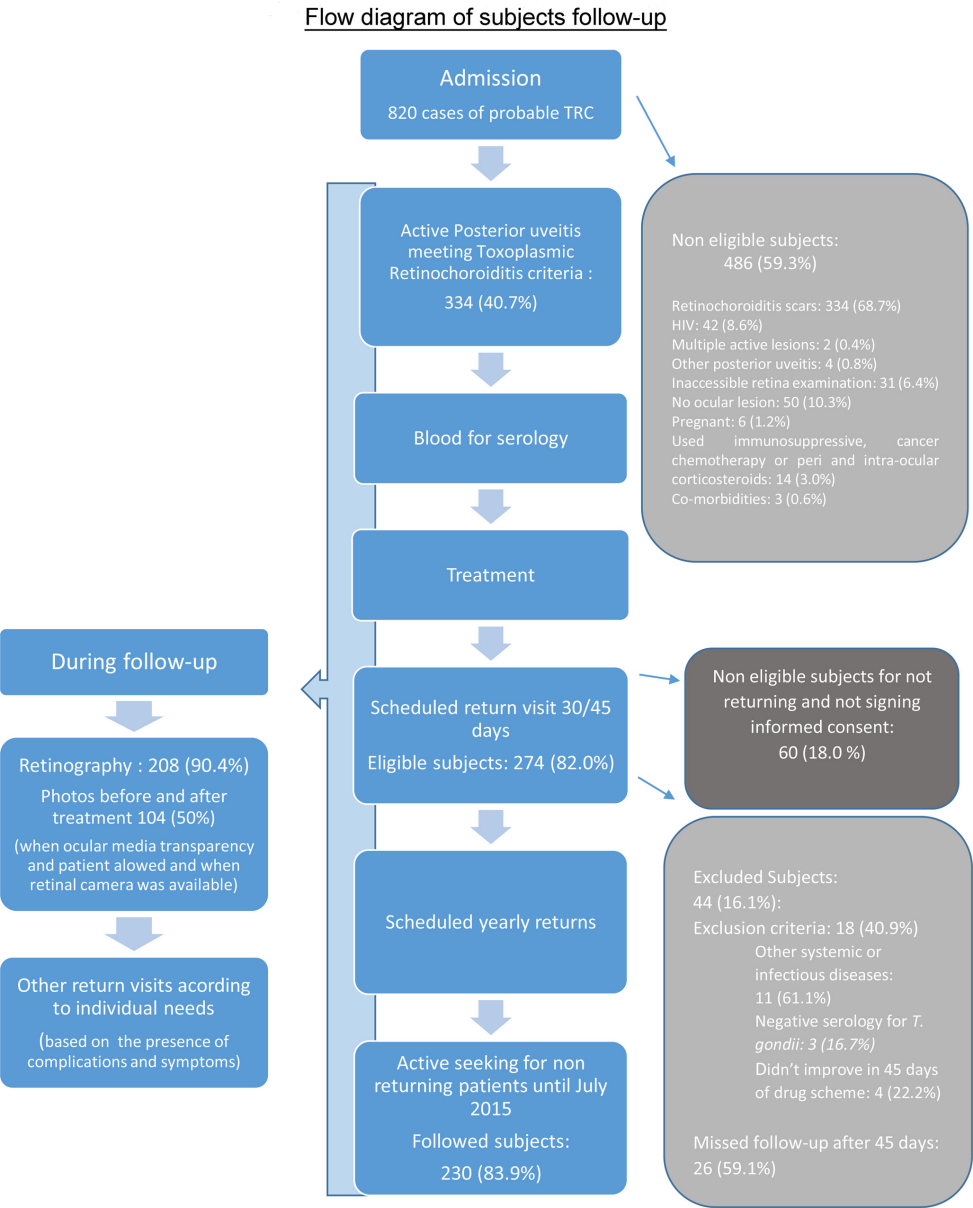
Flow diagram of subjects follow-up. Data analysis employed summary measures such as means and medians of quantitative variables and percentages for qualitative variables. To investigate the association between severe vision impairment and posterior segment complications, age, location of retinochoroidal lesion and recurrence during follow-up, the chi-square test was used and P-values <0.05 were considered significant.

All subjects with active retinochoroidal lesions, both in the initial active episode and in the subsequent reactivations, were treated for 30 to 45 days with standardized drug scheme for toxoplasmosis (sulfadiazine 4 x 1g daily, pyrimethamine 25–50 mg daily, folinic acid 15 mg every other day and prednisone 0.5 to 1 mg / kg / day as the initial dose followed by gradual tapper off) for 4 weeks or alternatively with 800mg-160mg 12/12h sulfametoxazole/ trimethoprim (SMZ-TMP) and prednisone or clindamycin (4 x 300 mg daily) replacing sulfadiazine in case of allergy. Serologic tests for toxoplasmosis were performed at the Toxoplasmosis Laboratory of IOC-Fiocruz by immunoenzymatic test (ELISA) and indirect immunofluorescence assay (RIFI). Follow-ups through July 2015 were used in the result analysis, which was performed as planned (Fig 5).

## Results

We prospectively followed 230 subjects from January 2010 until July 2015, 118 (51.3%) men and 112 (48.7 women for periods ranging from 269 to 1976 days, mean 1060 days. (S1 Chart)

All subjects included in the study lived in the State of Rio de Janeiro and only one reported nationality other than Brazilian. Ages ranged from 14 to 77 years old (mean = 32.4, SD = 11.4) distributed as shown in Table 1.

**Table 1:** Age distribution of 230 subjects with toxoplasma retinochoroiditis observed at INI.

Age in years	N/ %	Cum %
14–19	22/ 9.6	9.6
20–29	85/ 37.0	46.6
30–39	73/ 31.7	78.3
>40	50/ 21.7	100.0
Total	230/100.0	100.0

At the beginning of the study 52 (22.6%) patients with primary retinochoroidal lesions and 178 (77.4%) with recurrent lesions were observed. In 91 of 178 (51.1%) individuals it was associated with one scar and in 87 (48.9%) individuals it was associated with more than one retinochoroiditis scar. There were 162 recurrence episodes in 104 (45.2%) subjects during follow-up, 53 episodes (32.7%) occurred within the first year, 53 (32.7%) in the second year and 56 (34.6%) in the subsequent years. Among 104 subjects who presented recurrence, 14 (13.4%) individuals had two episodes in the same year and 43 (41.7%) had more than one episode of recurrence throughout follow-up. Recurrences were observed away from previous scars in 20 (12.3%) of the 162 episodes. Recurrences were not observed in 19 of the 52 (36.5%) patients with a primary lesion during the studied period. At the beginning 177 of 230 (76.9%) individuals had unilateral disease and 9 (3.9%) developed retinochoroidal lesion in the otherwise healthy eye during follow-up.

Most subjects (68.7%) were between 20–39 years old and it was possible to determine the age of the first episode in only 83 (36.1%) patients. Subclinical new retinochoroidal lesions were detected in 23 of 162 (14.2%) recurrences episodes during the follow-up, by visualization of small active lesions in routine consultations in 3 cases (13.0%), by photographic documentation in 7 cases (30.5%) or description of the number of lesions 13 cases (56.5%). A hundred fifty nine (69.1%) individuals could be identified with evidences of a subclinical scenario of ocular toxoplasmosis up to the moment they were examined in this study.

It was possible to retrospectively identify congenital toxoplasmosis in only one case (0.4%), and acute systemic symptomatic infection in three (1.3%) of 230 individuals; the time of first exposure for *T*. *gondii* was undeterminable in the remaining patients. Positive IgM serology for *T*. *gondii* was found in 21 (9.1%) individuals.

At least one central lesion (within the large vascular arcades) was observed in 172 (74.8%) subjects, and in 58 (25.2%) only peripheral lesions (outside the large vascular arcades) were seen. Ninety (52.3%) of these 172 patients had retinochoroidal lesions adjacent to the optic disc or in the macular area. Posterior segment complications attributable to TRC (described in Table 2) were observed in 73 of 230 (31.7%) patients and it was more frequent in subjects older than 40 years of age (p = 0.008). Five patients (2.2%) had diagnosis of outer punctate toxoplasmosis. Vasculitis, neuro-retinitis and perilesional neovascularization were observed respectively in 14 (6.1%), 3 (1.3%) and 4 (1.7%) of the 230 subjects and were not considered complications but clinical manifestations of ocular toxoplasmosis Vasculitis and perilesional neovascularization were seen in association with active exudative lesions before treatment and remission was observed in all cases after the standardized drug scheme used. Neuro-retinitis cases progressed to optic atrophy in 2 subjects. The four patients with retinal detachment were referred to surgery, as well as 3 subjects with epi-retinal membranes and 3 with residual vitreous opacities. Criteria of surgery recommendation for macular hole, epi-retinal membranes and macular pucker was based on the presence of retinal traction or visual acuity worse than 20/100. Residual vitreous opacities were defined as those seen until the last visit of the patient follow-up and were only referred to surgery if visual acuity remained worse than 20/100, at least 6 months after the last active episode. When these complications were associated with macular scars surgery was discussed with the patient regarding visual prognosis. Peripheral retinal tear was referred to treatment with laser photocoagulation, retinal hemorrhages and vitreous hemorrhage were managed conservatively with spontaneous resolution. Subjects with clinically evident macular edema that persisted after standardized drug scheme for toxoplasmosis were monitored and resolution observed within 3 months after the active episode.

**Table 2:** Complications of posterior segment attributable to toxoplasma retinochoroiditis in 73 of 230 subjects followed at the INI.

	N/%
Residual vitreous opacity	37 (43.5%)
Epi-retinal membrane	23 (27.1%)
Macular pucker	8 (9.4%)
Retinal Detachment	4 (4.7%)
Retinal hemorrhage	4 (4.7%)
Persistent macular edema	3 (3.5%)
Optic atrophy	2 (2.3%)
Macular hole	1 (1.2%)
Retinal tear	1 (1.2%)
Vitreous hemorrhage	1 (1.2%)
Sub-retinal fibrosis	1 (1.2%)
Total	85 (100%)

Two hundred and eight (90.4%) followed subjects initiated treatment of acute episodes with a standardized scheme for toxoplasmosis with sulfadiazine, pyrimethamine, prednisone and folinic acid, 15 subjects with SMZ-TMP (6.5%) and 7 (3.0%) had allergic reactions that justified the replacement of sulfadiazine by clindamycin and 11 (4.8%) subjects discontinued treatment without medical advice due to side effects of medications. Discontinuation of the treatment was not associated with recurrences (p = 0.76) or presence of complications (p = 0.99).

At the end of the follow-up 122 (53.0%) subjects had a decrease in visual acuity attributable to after-effects of the posterior uveitis (VA <20/30), 40 of 230 subjects (17.4%) had severe vision impairment (VA = 20/200 or worse) in the affected eye but in only 1 of 62 (1.6%) patients with bilateral lesions the visual acuity was 20/200 or worse in both eyes. There was an association between severe vision impairment and retinochoroidal location, complications and recurrences during follow up but not with age >40 years (Table 3).

**Table 3:** Factors related do severe vision loss in 230 subjects with ocular toxoplasmosis (univariate analysis).

	Severe vision impairment		
	Yes	No	p value
	N = 40	N = 190	
	(%)	(%)	
Posterior segment complications (N = 73)	21 (52.5)	52 (27.4)	0.0035
Age >40 years old (N = 50)	12 (30.0)	38 (20.0)	0.236
Adjacent to optic nerve and macular lesion (N = 90)	27 (67.5)	63 (33.2)	0.00011
Recurrence during follow-up (N = 104)	27 (67.5)	77 (40.5)	0.0032

## Discussion

Clinical manifestations of toxoplasmic retinochoroiditis have been described extensively in the literature, however the vast majority of the studies involve subjects treated at different centers, with different therapeutic regimens, with short follow-ups or retrospectively analyzed.[13–17] This study prospectively followed 230 subjects from a single center and evaluated in a standardized manner. Monitoring of asymptomatic subjects involved active search and persistent calls especially after the first year of follow-up which provided the detection of subclinical episodes of recurrence during follow-up. We observed 159 (69.1%) subjects with evidence of asymptomatic episodes up to the moment they were examined in the study, this shows that the disease can be subclinical in many individuals. This data was obtained by observing subclinical episodes during follow up and by comparing the number of retinochoroidal scars in the first visit of the study with the number of episodes referred by the patients. We cannot estimate how many of them had congenital or early childhood lesions that could justify the absence of previous history. Other possible causes of this observed phenomenon is the existence of previous vitreous opacities or vision impairment, which could make new symptoms more difficult to be perceived by the patient. Finding high rates of asymptomatic lesions may denote an underestimation of the frequency of the disease in individuals infected with *T*. *gondii*, a benign aspect due to the low morbidity associated with many lesions, but a hidden danger, with indefinite prognosis in patients in whom a retinochoroiditis scar is randomly diagnosed in clinical practice. This demonstrates the importance of studying risk factors associated with the recurrence of ocular toxoplasmosis in order to identify which individuals are more susceptible to the disease and devise strategies for monitoring and prevention.

Active seeking of asymptomatic subjects during follow-up was a strategy used to avoid the bias of monitoring just patients who had more severe and recurrent disease. Unfortunately, it was not possible to separate individuals in congenital and acquired ocular toxoplasmosis groups because in only 4 (1.7%) of 230 cases the moment of first exposure was certain. Serology for toxoplasmosis with positive IgM antibodies was detected in 21 (9.1%) followed subjects, assuming acquired disease in such cases. The low percentage of serologic confirmed acquired cases does not allow inferences about the mode of infection acquisition by the entire population. The predominance of quiescent retinochoroidal scars regarding the frequency of primary lesions (22.6%) has been described previously.[1,13,18,19] Individuals with primary retinochoroiditis lesion and no recurrence during the follow-up were included after negative results for HIV and syphilis tests and if they responded to specific drug treatment for toxoplasmosis, meeting these criteria, they were considered highly probable of TRC.

There are several sources of potential bias in this study. First of all, we cannot completely rule out mild tuberculous, histoplasmosis and herpes uveitis especially in those subjects where recurrence was not observed. Other published studies often have the same limitation as these diagnosis are frequently made on exclusion or presumed basis and ocular toxoplasmosis is only confirmed by intra-ocular fluid analyses eventually.[13,20,21] On the other hand, clinical evolution and the observed fundoscopic characteristics speaks favorably towards TRC diagnosis, cases with inaccessible retinal examination were considered ineligible and those with multiple active lesions were excluded as they lacked laboratory confirmation with intra-ocular fluid examination. Subclinical episodes were described based on sequenced retinographies but also by counting lesions, which brings a remote possibility of missed lesions in prior examinations being wrongly considered. What minimizes this possibility was the standardized indirect ophthalmoscopy examination performed by an uveitis experienced staff. Another aspect that should be considered concerning the subclinical episodes is that 3 subjects were diagnosed with active retinochoroidal lesions without new symptoms, these episodes may have been diagnosed before symptoms arose because of the programmed schedule of visits. The frequency of complications should also be carefully analyzed. If OCT and fluorescein angiography were routinely performed the complications rate could have been higher. Unfortunately, these ancillary tests were only requested to confirm clinical diagnosis of complications and investigation of vision loss and were performed in other health units because they were not available in our service by that time. For this reason they could not be included in our study protocol.

The studied population showed a homogeneous distribution in relation to gender, and a larger number of cases among 20–29 years and 30–39 years (36.9% and 31.7% respectively) was also observed in other series described in literature. [1,13,22] One hundred and sixty two episodes of recurrence in 104 (45.2%) patients were observed during follow-up, and this high recurrence rate was also observed in other studies.[17–19,22–24] It was found that 53 (32.7%) episodes of recurrence took place within the first year of follow-up and other 53 (32.7%) in the second year, which suggest a high risk of recurrence in the first two years after an episode of active retinochoroiditis, however, this should be confirmed by other statistical analysis specially concerning survival time. The clustering pattern of recurrences in TRC has been described previously and although the causes of reactivation are not completely understood, factors such as age and host defenses have been proposed to influence this aspect of the disease.[25]

If we consider that other publications involving subjects treated with different short term therapeutic regimens also had high recurrence rates, it can be assumed that the treatment used in this series of cases may have little influence on the frequency of recurrences, but this should be assessed by further studies, also concerning the influence of inflammation on recurrences.[13,16,17,26]

Some representative series of outbreaks, such as those occurred in Canada and India, found much lower rates of recurrence; this may be related to the strain of *T*. *gondii* involved and the particular characteristics of the population evaluated, such as the time of acquiring the disease and even a possible re-infection as the cause of recurrence.[14,27]

The occurrence of severe vision impairment in the affected eye was observed in 40 (17.4%) of 230 patients, a slightly lower incidence of blindness when compared to other series.[13] As expected, severe visual impairment was associated with the location of the retinochoroidal lesions, recurrence and with the occurrence of complications. The association of individuals with more than 40 years of age and complications suggests a tendency of increased disease severity in patients of this age group that requires further studies especially concerning recurrences in this population. The rate of retinal detachment, the overall rate of complications and vision impairment was slightly smaller than that previously described in other series, which may be eventually related with the treatment regimen used but also with the exclusion of non-confirmed severe cases that lack intra-ocular fluid examination.[13,28,29] Several studies in the literature consider the visual outcome and number of lesions as a measure to assess the effectiveness of therapeutic regimens and prevention strategies, and if this is done without taking into consideration the location of the lesion, erroneous conclusions may be drawn, as the location of the lesion is not treatment related.[6,30]

Involvement of the otherwise healthy eye developed in only 9 (3.9%) subjects who started the study with unilateral disease and can be considered a rare event. The frequency of bilateral retinochoroidal scars has been described in 22–44% of subjects in other series, and it is similar to the frequency found in our study.[1,13,19,22]

In summary, recurrences were observed in 45.2% of the followed subjects and the severe vision impairment found in less than 20% of subjects, and it was associated with the location of the retinochoroidal scar, recurrences and posterior segment complications.

High recurrence rates were observed after an active episode of TRC in this case series. Subclinical episodes in adults were observed in this studied population and can be a cause of underestimation of recurrences in retrospective studies. It is crucial to consider the location of the retinochoroidal lesion in studies analyzing the visual outcome as a measure of the effectiveness of treatment and prevention strategies.

## Supporting Information

S1 Checklist Strobe checklist. (DOC)

S1 Chart Toxoplasma retinochoroiditis subjects and its follow-up time. Each bullet represents a TRC subject.
(JPG)

S1 Text Data analyses. (PDF)

## References

[1] Friedmann CT, Knox DL (1969) Variations in recurrent active toxoplasmic retinochoroiditis. Arch Ophthalmol 81: 481–493. 5777756 10.1001/archopht.1969.00990010483005

[2] Holland GN (2003) Ocular toxoplasmosis: a global reassessment. Part I: epidemiology and course of disease. Am J Ophthalmol 136: 973–988. 14644206 10.1016/j.ajo.2003.09.040

[3] Holland GN, Lewis KG, O'Connor GR (2002) Ocular toxoplasmosis: a 50th anniversary tribute to the contributions of Helenor Campbell Wilder Foerster. Arch Ophthalmol 120: 1081–1084. 12149063 10.1001/archopht.120.8.1081

[4] Butler NJ, Furtado JM, Winthrop KL, Smith JR (2013) Ocular toxoplasmosis II: clinical features, pathology and management. Clin Experiment Ophthalmol 41: 95–108. 10.1111/j.1442-9071.2012.02838.x 22712598 PMC4028599

[5] Garweg JG, Stanford MR (2013) Therapy for ocular toxoplasmosis—the future. Ocul Immunol Inflamm 21: 300–305. 10.3109/09273948.2013.779724 23617277

[6] Harrell M, Carvounis PE (2014) Current treatment of toxoplasma retinochoroiditis: an evidence-based review. J Ophthalmol 2014: 273506 10.1155/2014/273506 25197557 PMC4147351

[7] Holland GN (2004) Ocular toxoplasmosis: a global reassessment. Part II: disease manifestations and management. Am J Ophthalmol 137: 1–17. 14700638

[8] Vaudaux JD, Muccioli C, James ER, Silveira C, Magargal SL, et al (2010) Identification of an atypical strain of toxoplasma gondii as the cause of a waterborne outbreak of toxoplasmosis in Santa Isabel do Ivai, Brazil. J Infect Dis 202: 1226–1233. 10.1086/656397 20836703 PMC5718918

[9] Verma SK, Ajzenberg D, Rivera-Sanchez A, Su C, Dubey JP (2015) Genetic characterization of Toxoplasma gondii isolates from Portugal, Austria and Israel reveals higher genetic variability within the type II lineage. Parasitology: 1–10. 10.1017/S0031182015000050 25677825

[10] Glasner PD, Silveira C, Kruszon-Moran D, Martins MC, Burnier Junior M, et al (1992) An unusually high prevalence of ocular toxoplasmosis in southern Brazil. Am J Ophthalmol 114: 136–144. 1642287 10.1016/s0002-9394(14)73976-5

[11] Holland G, O’Connor GR, Belfort R Jr, Remington JS. Toxoplasmosis In: Pepose JS, Holland GN, Wilhelmus KR, (1996) Ocular Infection & Immunity. St Louis, Missouri: Mosby-Year Book, Inc,:1183–1223.

[12] World Health Organization International Statistical Classification of Diseases and Related Health Problems pp. 10th revision Current version Version for 2003 Chapter VII H2054 Blindness and low vision.

[13] Bosch-Driessen LE, Berendschot TT, Ongkosuwito JV, Rothova A (2002) Ocular toxoplasmosis: clinical features and prognosis of 154 patients. Ophthalmology 109: 869–878. 11986090 10.1016/s0161-6420(02)00990-9

[14] Burnett AJ, Shortt SG, Isaac-Renton J, King A, Werker D, et al (1998) Multiple cases of acquired toxoplasmosis retinitis presenting in an outbreak. Ophthalmology 105: 1032–1037. 9627653 10.1016/S0161-6420(98)96004-3

[15] de-la-Torre A, Rios-Cadavid AC, Cardozo-Garcia CM, Gomez-Marin JE (2009) Frequency and factors associated with recurrences of ocular toxoplasmosis in a referral centre in Colombia. Br J Ophthalmol 93: 1001–1004. 10.1136/bjo.2008.155861 19429576

[16] Accorinti M, Bruscolini A, Pirraglia MP, Liverani M, Caggiano C (2009) Toxoplasmic retinochoroiditis in an Italian referral center. Eur J Ophthalmol 19: 824–830. 19787604 10.1177/112067210901900522

[17] Garweg JG, Scherrer JN, Halberstadt M (2008) Recurrence characteristics in European patients with ocular toxoplasmosis. Br J Ophthalmol 92: 1253–1256. 10.1136/bjo.2007.123661 18211930

[18] O'Connor GR (1974) Manifestations and management of ocular toxoplasmosis. Bull N Y Acad Med 50: 192–210. 4521729 PMC1749344

[19] Rothova A (1993) Ocular involvement in toxoplasmosis. Br J Ophthalmol 77: 371–377. 8318486 10.1136/bjo.77.6.371 PMC504534

[20] Amaro MH, Muccioli C, Abreu MT (2007) Ocular histoplasmosis-like syndrome: a report from a nonendemic area. Arq Bras Oftalmol 70: 577–580. 17906749 10.1590/s0004-27492007000400003

[21] Shakarchi FI (2015) Ocular tuberculosis: current perspectives. Clin Ophthalmol 9: 2223–2227. 10.2147/OPTH.S65254 26648690 PMC4664543

[22] Gilbert RE, Dunn DT, Lightman S, Murray PI, Pavesio CE, et al (1999) Incidence of symptomatic toxoplasma eye disease: aetiology and public health implications. Epidemiol Infect 123: 283–289. 10579449 10.1017/s0950268899002800 PMC2810761

[23] Holland GN (2009) Ocular toxoplasmosis: the influence of patient age. Mem Inst Oswaldo Cruz 104: 351–357. 19430663 10.1590/s0074-02762009000200031

[24] Bosch-Driessen EH, Rothova A (1999) Recurrent ocular disease in postnatally acquired toxoplasmosis. Am J Ophthalmol 128: 421–425. 10577582 10.1016/s0002-9394(99)00271-8

[25] Holland GN, Crespi CM, ten Dam-van Loon N, Charonis AC, Yu F, et al (2008) Analysis of recurrence patterns associated with toxoplasmic retinochoroiditis. Am J Ophthalmol 145: 1007–1013. 10.1016/j.ajo.2008.01.023 18343351

[26] Reich M, Becker MD, Mackensen F (2016) Influence of drug therapy on the risk of recurrence of ocular toxoplasmosis. Br J Ophthalmol 100: 195–199. 10.1136/bjophthalmol-2015-306650 26163541

[27] Balasundaram MB, Andavar R, Palaniswamy M, Venkatapathy N (2010) Outbreak of acquired ocular toxoplasmosis involving 248 patients. Arch Ophthalmol 128: 28–32. 10.1001/archophthalmol.2009.354 20065213

[28] Bosch-Driessen LH, Karimi S, Stilma JS, Rothova A (2000) Retinal detachment in ocular toxoplasmosis. Ophthalmology 107: 36–40. 10647716 10.1016/s0161-6420(99)00013-5

[29] Faridi A, Yeh S, Suhler EB, Smith JR, Flaxel CJ (2015) Retinal detachment associated with ocular toxoplasmosis. Retina 35: 358–363. 10.1097/IAE.0000000000000279 25127047

[30] Felix JP, Lira RP, Zacchia RS, Toribio JM, Nascimento MA, et al (2014) Trimethoprim-sulfamethoxazole versus placebo to reduce the risk of recurrences of Toxoplasma gondii retinochoroiditis: randomized controlled clinical trial. Am J Ophthalmol 157: 762–766.e761. 10.1016/j.ajo.2013.12.022 24388839

