# Supplementary material for: Toxoplasmic Retinochoroiditis: Clinical Characteristics and Visual Outcome in a Prospective Study
Source: PLoS Negl Trop Dis. 2016 May 2;10(5):e0004685. doi: 10.1371/journal.pntd.0004685 (PMC4852945; doi:10.1371/journal.pntd.0004685)
Supplement: S1 Checklist — (DOC) [file pntd.0004685.s001.doc]

STROBE Statement—checklist

**Ocular toxoplasmosis clinical characteristics and visual outcome in a prospective study**

|  | Item No | Recommendation |  |
| --- | --- | --- | --- |
| **Title and abstract** | 1 | (*a*) **Indicate the study’s design with a commonly used term in the title or the abstract**  *Toxoplasmic Retinochoroiditis: clinical characteristics and visual outcome in a prospective study* | Yes |
|  |  | (*b*) **Provide in the abstract an informative and balanced summary of what was done and what was found** | Yes  Page 2 |
| Introduction | | |  |
| Background/rationale | 2 | **Explain the scientific background and rationale for the investigation being reported**  *Brazil hosts the highest prevalence ever described for ocular toxoplasmosis with possible preponderance of different strains from those that predominate in Europe and North America, this alone would possibly justify clinical differences and long term outcome* | Page 3 |
| Objectives | 3 | **State specific objectives, including any prespecified hypotheses**  *To observe outcomes of toxoplasma retinochoroiditis (TRC) and especially the causes of vision loss in affected patients is crucial to guide the analysis of prevention strategies and treatment.*  *(…)*  *The study herein presented describes the clinical characteristics of a series of prospectively followed cases of toxoplasma retinochoroiditis, analyzing the course of the disease, recurrence frequency and factors that may influence the visual prognosis*. | Page 3 |
| Methods | | |  |
| Study design | 4 | **Present key elements of study design early in the paper**  *A prospective observational and non-experimental study with 230 subjects with active TRC consulted at the outpatient unit of the Infectious Ophthalmology Laboratory of Evandro Chagas National Institute of Infectious Diseases (INI) was conducted after approval by the INI Ethics Committee (CAAE 0075.0.009.00011). These individuals were selected by the same ophthalmologist from 1912 patients examined between January 2010 and January 2014 of which 820 (42.9%) were referred as probable TRC. Two hundred seventy four individuals were considered eligible with diagnosis of active toxoplasma retinochoroiditis and no exclusion criteria at admission and agreed to participate in the research signing an informed consent. Subjects between 14 and 18 years old who agreed to participate also signed an informed consent in conjunction with a parent or surrogate. Twenty six subjects (9.5%) missed follow-up consultation and 18 (6.5%) met exclusion criteria during the follow-up, though were not included in the analysis. All procedures performed with the subjects were approved by the INI Ethic Committee and followed the standard routine of the outpatient unit for ocular toxoplasmosis***.** | Page  3-4 |
| Setting | 5 | **Describe the setting, locations, and relevant dates, including periods of recruitment, exposure, follow-up, and data collection**  *These individuals were selected by the same ophthalmologist from 1912 patients examined between January 2010 and January 2014 of which 820 (42.9%) were referred as probable TRC. Two hundred seventy four individuals were considered eligible with diagnosis of active toxoplasma retinochoroiditis and no exclusion criteria at admission and agreed to participate in the research signing an informed consent. Subjects between 14 and 18 years old who agreed to participate also signed an informed consent in conjunction with a parent or surrogate. Twenty six subjects (9.5%) missed follow-up consultation and 18 (6.5%) met exclusion criteria during the follow-up, though were not included in the analysis.*  *(…)*  *We prospectively followed 230 patients from January 2010 until July 2015 (…)* | Page 3,4-7 |
| Participants | 6 | (***a*) *Cohort study*—Give the eligibility criteria, and the sources and methods of selection of participants. Describe methods of follow-up**  *(…)A prospective observational and non-experimental study with 230 subjects with active TRC consulted at the outpatient unit of the Infectious Ophthalmology Laboratory of Evandro Chagas National Institute of Infectious Diseases (INI) was conducted after approval by the INI Ethics Committee (CAAE 0075.0.009.00011). These individuals were selected by the same ophthalmologist from 1912 patients examined between January 2010 and January 2014 of which 820 (42.9%) were referred as probable TRC. Two hundred seventy four individuals were considered eligible with diagnosis of active toxoplasma retinochoroiditis and no exclusion criteria at admission and agreed to participate in the research signing an informed consent. Subjects between 14 and 18 years old who agreed to participate also signed an informed consent in conjunction with a parent or surrogate. Twenty six subjects (9.5%) missed follow-up consultation and 18 (6.5%) met exclusion criteria during the follow-up, though were not included in the analysis. All procedures performed with the subjects were approved by the INI Ethic Committee and followed the standard routine of the outpatient unit for ocular toxoplasmosis****.***  ***Follow-up***  *Follow-up visits were scheduled within 30 or 45 days and every year after the initial consultation. Yearly returns were programmed for every subject as well as individual consultations according to the presence of complications. Subjects were instructed to seek the Infectious Ophthalmology Laboratory of INI at any time in case of eye symptoms, especially blurred vision, red eye or floaters. Subjects who did not return on the scheduled dates were contacted by phone or mail and were only included in the study if examined within 45 days after the initial consultation and followed for at least one more visit in a minimum of 8 months. A minimal of three consultations was required to consider the patient followed, one for admission, the second within 45 days after the first one and the third between 8 months after the first visit and July 2015.* *Retinography was performed for assessment of retinal status before and after treatment whenever the transparency of the ocular media and the patient allowed and depending on the availability of the retinal camera.*  *Case-control study*—Give the eligibility criteria, and the sources and methods of case ascertainment and control selection. Give the rationale for the choice of cases and controls  *NA*  *Cross-sectional study*—Give the eligibility criteria, and the sources and methods of selection of participants  *NA* | Yes  Page 3-6 |
|  |  | (*b*) *Cohort study*—For matched studies, give matching criteria and number of exposed and unexposed  *Case-control study*—For matched studies, give matching criteria and the number of controls per case | NA |
| Variables | 7 | **Clearly define all outcomes, exposures, predictors, potential confounders, and effect modifiers. Give diagnostic criteria, if applicable**  *For the purpose of this study, the diagnosis of active toxoplasmic retinochoroiditis was based on clinical criteria formulated by Holland et al in seropositive patients for T. gondii [11] Primary TRC was defined as creamy-white exudative focal retinochoroiditis not associated with retinochoroidal scars in either eye. Recurrent TRC was defined as a focal active retinochoroiditis associated with retinal scarring in the same or contralateral eye (figures 1, 2 , 3 and 4).[7]*  *Subclinical episodes were defined as new healed or active lesions observed in fundoscopic examination (by comparing the number of lesions described in the admission consultation or comparing retinographies) in subjects who didn’t exhibit new significant symptoms. Significant symptoms were defined as those referred spontaneously by the patient. Episodes of anterior segment inflammation in eyes with retinochoroidal scars had previously been described on patients with ocular toxoplasmosis and were not considered recurrences.[11] Subjects with co-morbidities such as chronic renal failure, systemic infections as AIDS, syphilis and tuberculosis were not considered eligible as well as those with diagnosed auto immune diseases. History of intravenous drugs use, cancer chemotherapy, immunosuppressive drugs or peri and intraocular steroids were considered exclusion criteria. Subjects with single and unilateral exudative retinochoroiditis with positive diagnostic tests for syphilis (VDRL and TPHA, imunoflocculation and hemagglutination for T. pallidum) and HIV 1 and 2 (rapid test and / or serology by ELISA) performed in the Laboratory of Immunology and Immunogenetics of the INI were also excluded. Individuals with single lesions who did not respond to treatment with the standardized drug scheme for toxoplasmosis during the first 45 days and who were pregnant during any recurrent episodes were also excluded, as well as subjects with multiple exudative lesions of retinochoroiditis.* | Page  3-4 |
| Data sources/ measurement | 8* | For each variable of interest, give sources of data and details of methods of assessment (measurement). Describe comparability of assessment methods if there is more than one group | *Page 3-4* |
| Bias | 9 | **Describe any efforts to address potential sources of bias**  *Active seeking of asymptomatic patients during follow-up was a strategy used to avoid the bias of monitoring just patients who had more severe and recurrent disease.*  *There are several sources of potential bias in this study. First of all, we cannot completely rule out mild tuberculous, histoplasmosis and herpes uveitis especially in those subjects that recurrence was not observed. Other published studies often have the same limitation as these diagnosis are frequently made on exclusion or presumed basis and ocular toxoplasmosis is only confirmed by intra-ocular fluid analyses eventually.[13,20,21] On the other hand, clinical evolution and the observed fundoscopic characteristics speaks favorably towards TRC diagnosis, cases with inaccessible retinal examination were considered ineligible and those with multiple active lesions were excluded as they lack laboratory confirmation with intra-ocular fluid examination. Subclinical episodes were described based on sequenced retinographies but also by counting lesions, what brings a remote possibility of missed lesions in prior examinations being wrongly considered. What minimizes this error possibility is the standardized indirect ophthalmoscopy examination performed by an uveitis experienced staff. Another aspect that should be considered concerning the subclinical episodes is that 3 subjects were diagnosed with active retinochoroidal lesions without new symptoms, this episodes may have been diagnosed before symptoms arise because of the programmed schedule of visits. The frequency of complications should also be carefully analyzed. If OCT and fluorescein angiography was routinely performed the complications rate could be higher. Unfortunately these procedures were only performed to confirm clinical diagnosis of complications and investigation of vision loss out of the study protocol, in other health units by the middle of 2014, when they were incorporated to the Infectious Ophthalmology Laboratory of Evandro Chagas National Institute of Infectious Diseases.*  *The rate of retinal detachment, the overall rate of complications and vision impairment was slightly smaller than that previously described in other series, which may be eventually related with the treatment regimen used but also with the exclusion of non-confirmed severe cases that lack intra-ocular fluid examination.[28,29]* | Page 12-15 |
| Study size | 10 | **Explain how the study size was arrived at**  *A prospective observational and non-experimental study with 230 subjects with active TRC consulted at the outpatient unit of the Infectious Ophthalmology Laboratory of Evandro Chagas National Institute of Infectious Diseases (INI) was conducted after approval by the INI Ethics Committee (CAAE 0075.0.009.00011). These individuals were selected by the same ophthalmologist from 1912 patients examined between January 2010 and January 2014 of which 820 (42.9%) were referred as probable TRC. Two hundred seventy four individuals were considered eligible with diagnosis of active toxoplasma retinochoroiditis and no exclusion criteria at admission and agreed to participate in the research signing an informed consent. Subjects between 14 and 18 years old who agreed to participate also signed an informed consent in conjunction with a parent or surrogate. Twenty six subjects (9.5%) missed follow-up consultation and 18 (6.5%) met exclusion criteria during the follow-up, though were not included in the analysis.*  *See also flow diagram of subjects follow-up* | Page 3-4 |
| Quantitative variables | 11 | **Explain how quantitative variables were handled in the analyses. If applicable, describe which groupings were chosen and why**  *Data analysis employed summary measures such as means and medians of quantitative variables and percentages for qualitative variables. To investigate the association between severe visual loss and posterior segment complications, age, location of retinochoroidal lesion and recurrence during follow-up, the chi-square test was used and P-values <0.05 were considered significant.* | Page  6-7 |
| Statistical methods | 12 | ***(*a) Describe all statistical methods, including those used to control for confounding**  *Data analysis employed summary measures such as means and medians of quantitative variables and percentages for qualitative variables. To investigate the association between severe visual loss and posterior segment complications, age, location of retinochoroidal lesion and recurrence during follow-up, the chi-square test was used and P-values <0.05 were considered significant.* | Page 6-7 |
|  |  | (*b*) Describe any methods used to examine subgroups and interactions | NA |
|  |  | (*c*) Explain how missing data were addressed |  |
|  |  | **(*d*) *Cohort study*—If applicable, explain how loss to follow-up was addressed**  *Two hundred seventy four individuals were considered eligible with diagnosis of active toxoplasma retinochoroiditis and no exclusion criteria at admission and agreed to participate in the research signing an informed consent. Patients between 14 and 18 years old who agreed to participate also signed an informed consent in conjunction with a parent or surrogate. Twenty six patients (9.5%) missed follow-up consultation and 18 (6.5%) met exclusion criteria during the follow-up, though* ***were not included in the analysis***  *Case-control study*—If applicable, explain how matching of cases and controls was addressed  *Cross-sectional study*—If applicable, describe analytical methods taking account of sampling strategy | Page 4 |
|  |  | (*e*) Describe any sensitivity analyses | NA |

Continued on next page

| Results | | |  |
| --- | --- | --- | --- |
| Participants | 13* | (a) Report numbers of individuals at each stage of study—eg numbers potentially eligible, examined for eligibility, confirmed eligible, included in the study, completing follow-up, and analysed | Page 4 and Flow chart |
|  |  | (b) Give reasons for non-participation at each stage | Page 4 |
|  |  | (c) Consider use of a flow diagram |  |
| Descriptive data | 14* | **(a) Give characteristics of study participants (eg demographic, clinical, social) and information on exposures and potential confounders**  **(b) Indicate number of participants with missing data for each variable of interest**  **(c) *Cohort study*—Summarise follow-up time (eg, average and total amount)**  *We prospectively followed 230 patients from January 2010 until July 2015, 118 (51.3%) men and 112 (48.7 women for periods ranging from 269 to 1976 days, mean 1060 days. All patients included in the study reported to live in the State of Rio de Janeiro and only one reported nationality other than Brazilian. Ages ranged from 14 to 77 years old (mean = 32.4, SD = 11.4) distributed as shown in table 1.* | Page 7 |
| Outcome data | 15* | ***Cohort study*—Report numbers of outcome events or summary measures over time**  *There were 162 recurrence episodes in 104 (45.2%) patients during follow-up, 53 episodes (32.7%) occurred within the first year, 53 (32.7%) in the second year and 56 (34.5%) in the subsequent years* | Page 8 |
|  |  | *Case-control study—*Report numbers in each exposure category, or summary measures of exposure | NA |
|  |  | *Cross-sectional study—*Report numbers of outcome events or summary measures | NA |
| Main results | 16 | (*a*) Give unadjusted estimates and, if applicable, confounder-adjusted estimates and their precision (eg, 95% confidence interval). Make clear which confounders were adjusted for and why they were included | Page 7-11 |
|  |  | (*b*) Report category boundaries when continuous variables were categorized  *See Table 1* |  |
|  |  | (*c*) If relevant, consider translating estimates of relative risk into absolute risk for a meaningful time period | NA |
| Other analyses | 17 | Report other analyses done—eg analyses of subgroups and interactions, and sensitivity analyses | NA |
| Discussion | | |  |
| Key results | 18 | **Summarise key results with reference to study objectives**  *In summary, recurrences were observed in 45.2% of the followed subjects and the severe visual impairment found in less than 20% of subjects, and it was associated with the location of the retinochoroidal scar, recurrences and posterior segment complications.*  *High recurrence rates were observed after an active episode of TRC in this case series. Subclinical episodes in adults were observed in this studied population and can be a cause of underestimation of recurrences in retrospective studies. It is crucial to consider the location of the retinochoroidal lesion in studies analyzing the visual outcome as a measure of the effectiveness of treatment and prevention strategies.* | Page 9-11 |
| Limitations | 19 | **Discuss limitations of the study, taking into account sources of potential bias or imprecision. Discuss both direction and magnitude of any potential bias**  *Lines 258-302.* | Page 11-12 |
| Interpretation | 20 | **Give a cautious overall interpretation of results considering objectives, limitations, multiplicity of analyses, results from similar studies, and other relevant evidence** | Page  11-12 |
| Generalisability | 21 | **Discuss the generalisability (external validity) of the study results** | Page 11-12 |
| Other information | | |  |
| Funding | 22 | **Give the source of funding and the role of the funders for the present study and, if applicable, for the original study on which the present article is based**  *This research received no specific grant from any funding agency in the public, commercial or not-for-profit sectors.* | NA |

*Give information separately for cases and controls in case-control studies and, if applicable, for exposed and unexposed groups in cohort and cross-sectional studies.

**Note:** An Explanation and Elaboration article discusses each checklist item and gives methodological background and published examples of transparent reporting. The STROBE checklist is best used in conjunction with this article (freely available on the Web sites of PLoS Medicine at http://www.plosmedicine.org/, Annals of Internal Medicine at http://www.annals.org/, and Epidemiology at http://www.epidem.com/). Information on the STROBE Initiative is available at www.strobe-statement.org.
