# Supplementary figures and images for: Toxoplasmic Retinochoroiditis: Clinical Characteristics and Visual Outcome in a Prospective Study
Source: PLoS Negl Trop Dis. 2016 May 2;10(5):e0004685. doi: 10.1371/journal.pntd.0004685 (PMC4852945; doi:10.1371/journal.pntd.0004685)

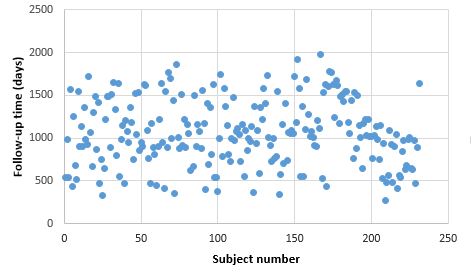

Supplement: S1 Chart — Each bullet represents a TRC subject. (JPG) [file pntd.0004685.s002.JPG]
