# Supplementary material for: Toxoplasmic Retinochoroiditis: Clinical Characteristics and Visual Outcome in a Prospective Study
Source: PLoS Negl Trop Dis. 2016 May 2;10(5):e0004685. doi: 10.1371/journal.pntd.0004685 (PMC4852945; doi:10.1371/journal.pntd.0004685)
Supplement: S1 Text — (PDF) [file pntd.0004685.s003.pdf]

Dashboard Output

Current data source: **Microsoft Excel Workbook: D:\In english- planilha analise acompanhamento.xlsx**
Record count: **230** *(Deleted records excluded) Date:* **30/09/2015 11:44**

Frequency

Frequency variable: **sex**
Include missing: **False**

sex	Frequency	Percent	Cum. Percent	Exact 95% LCL	Exact 95% LCL
F	112	48,70%	48,70%	42,07%	55,35%
M	118	51,30%	100,00%	44,65%	57,93%
**TOTAL**	**230**	**100,00%**	**100,00%**		

Means

Main variable: **follow-up-days**

follow-up-days											
	Obs	Total	Mean	Var	Std Dev	Min	25%	Median	75%	Max	Mode
follow-up-days	230	243817	1060,0739	147069,2391	383,4961	269,0000	781,0000	1029,0000	1369,0000	1976,0000	1358,0000

Means

Main variable: **age#years**

age#years											
	Obs	Total	Mean	Var	Std Dev	Min	25%	Median	75%	Max	Mode
age#years	230	7445,9165	32,3735	129,5981	11,3841	14,0507	23,8604	30,9747	38,5708	76,7447	20,6653

Crosstabulation (MxN, 2x2)

Main variable: **severe visual loss**
Crosstab variable: **age category**
Include missing: **False**

	age category		
severe visual loss	39 or less	40 years or more	Total
yes	28	12	40
Row %	70,00%	30,00%	100,00%
Col %	15,56%	24,00%	17,39%
no	152	38	190
Row %	80,00%	20,00%	100,00%
Col %	84,44%	76,00%	82,61%
Total	180	50	230
Row %	78,26%	21,74%	100,00%
Col %	100,00%	100,00%	100,00%

Single Table Analysis

	Point	95% Confidence Interval	
	Estimate	Lower	Upper
PARAMETERS: Odds-based			
Odds Ratio (cross product)	0,5833	0,2717	1,2523 (T)
Odds Ratio (MLE)	0,5848	0,2739	1,2924 (M)
		0,2586	1,3836 (F)
PARAMETERS: Risk-based			
Risk Ratio (RR)	0,8750	0,7057	1,0849 (T)
Risk Difference (RD%)	-10,0000	-25,2982	5,2982 (T)
(T=Taylor series; C=Cornfield; M=Mid-P; F=Fisher Exact)			
STATISTICAL TESTS	Chi-square	1-tailed p	2-tailed p
Chi-square - uncorrected	1,9422		0,1634274519
Chi-square - Mantel-Haenszel	1,9338		0,1643457291
Chi-square - corrected (Yates)	1,3989		0,2369054077
Mid-p exact		0,0889235894	
Fisher exact 1-tailed		0,1199732137	0,2045073301

Crosstabulation (MxN, 2x2)

Main variable: **severe visual loss**
Crosstab variable: **recurrence**
Include missing: **False**

	recurrence		
severe visual loss	yes	no	Total
yes	27	13	40
Row %	67,50%	32,50%	100,00%
Col %	25,96%	10,32%	17,39%
no	77	113	190
Row %	40,53%	59,47%	100,00%
Col %	74,04%	89,68%	82,61%
Total	104	126	230
Row %	45,22%	54,78%	100,00%
Col %	100,00%	100,00%	100,00%

Single Table Analysis

	Point	95% Confidence Interval	
	Estimate	Lower	Upper
PARAMETERS: Odds-based			
Odds Ratio (cross product)	3,0480	1,4802	6,2761 (T)
Odds Ratio (MLE)	3,0329	1,4837	6,4198 (M)
		1,4088	6,8306 (F)
PARAMETERS: Risk-based			
Risk Ratio (RR)	1,6656	1,2645	2,1939 (T)
Risk Difference (RD%)	26,9737	10,8671	43,0802 (T)
(T=Taylor series; C=Cornfield; M=Mid-P; F=Fisher Exact)			
STATISTICAL TESTS	Chi-square	1-tailed p	2-tailed p
Chi-square - uncorrected	9,7055		0,0018371696
Chi-square - Mantel-Haenszel	9,6633		0,0018798503
Chi-square - corrected (Yates)	8,6471		0,0032757584
Mid-p exact		0,0010470482	
Fisher exact 1-tailed		0,0016132200	0,0026745879

Crosstabulation (MxN, 2x2)

Main variable: **severe visual loss**
Crosstab variable: **peridiscal#macular**
Include missing: **False**

	peridiscal#macular		
severe visual loss	yes	no	Total
yes<br>Row %<br>Col %	27<br>67,50%<br>30,00%	13<br>32,50%<br>9,29%	40<br>100,00%<br>17,39%
no<br>Row %<br>Col %	63<br>33,16%<br>70,00%	127<br>66,84%<br>90,71%	190<br>100,00%<br>82,61%
Total<br>Row %<br>Col %	90<br>39,13%<br>100,00%	140<br>60,87%<br>100,00%	230<br>100,00%<br>100,00%

Single Table Analysis

	Point	95% Confidence Interval	
	Estimate	Lower	Upper
PARAMETERS: Odds-based			
Odds Ratio (cross product)	4,1868	2,0230	8,6648 (T)
Odds Ratio (MLE)	4,1587	2,0246	8,8471 (M)
		1,9212	9,4199 (F)
PARAMETERS: Risk-based			
Risk Ratio (RR)	2,0357	1,5157	2,7341 (T)
Risk Difference (RD%)	34,3421	18,3577	50,3265 (T)
(T=Taylor series; C=Cornfield; M=Mid-P; F=Fisher Exact)			
**STATISTICAL TESTS**	**Chi-square**	**1-tailed p**	**2-tailed p**
Chi-square - uncorrected	16,3616		0,0000523360
Chi-square - Mantel-Haenszel	16,2904		0,0000543379
Chi-square - corrected (Yates)	14,9515		0,0001103103
Mid-p exact		0,0000393699	
Fisher exact 1-tailed		0,0000645072	0,0000749443

Crosstabulation (MxN, 2x2)

Main variable: **severe visual loss**
Crosstab variable: **complications**
Include missing: **False**

	complications		
severe visual loss	yes	no	Total
yes	21	19	40
Row %	52,50%	47,50%	100,00%
Col %	28,77%	12,10%	17,39%
no	52	138	190
Row %	27,37%	72,63%	100,00%
Col %	71,23%	87,90%	82,61%
Total	73	157	230
Row %	31,74%	68,26%	100,00%
Col %	100,00%	100,00%	100,00%

Single Table Analysis

	Point	95% Confidence Interval	
	Estimate	Lower	Upper
PARAMETERS: Odds-based			
Odds Ratio (cross product)	2,9332	1,4599	5,8934 (T)
Odds Ratio (MLE)	2,9178	1,4451	5,9289 (M)
		1,3716	6,2576 (F)
PARAMETERS: Risk-based			
Risk Ratio (RR)	1,9183	1,3185	2,7908 (T)
Risk Difference (RD%)	25,1316	8,4076	41,8556 (T)
(T=Taylor series; C=Cornfield; M=Mid-P; F=Fisher Exact)			
**STATISTICAL TESTS**	**Chi-square**	**1-tailed p**	**2-tailed p**
Chi-square - uncorrected	9,6329		0,0019111934
Chi-square - Mantel-Haenszel	9,5910		0,0019552820
Chi-square - corrected (Yates)	8,5079		0,0035361453
Mid-p exact		0,0014539843	
Fisher exact 1-tailed		0,0022235913	0,0027476034

Crosstabulation (MxN, 2x2)

Main variable: **age category**
Crosstab variable: **complications**
Include missing: **False**

	complications		
age category	yes	no	Total
39 or less	49	131	180
Row %	27,22%	72,78%	100,00%
Col %	67,12%	83,44%	78,26%
40 years or more	24	26	50
Row %	48,00%	52,00%	100,00%
Col %	32,88%	16,56%	21,74%
Total	73	157	230
Row %	31,74%	68,26%	100,00%
Col %	100,00%	100,00%	100,00%

Single Table Analysis

	Point	95% Confidence Interval	
	Estimate	Lower	Upper
PARAMETERS: Odds-based			
Odds Ratio (cross product)	0,4052	0,2127	0,7720 (T)
Odds Ratio (MLE)	0,4070	0,2123	0,7797 (M)
		0,2028	0,8162 (F)
PARAMETERS: Risk-based			
Risk Ratio (RR)	0,5671	0,3900	0,8248 (T)
Risk Difference (RD%)	-20,7778	-36,0766	-5,4789 (T)
(T=Taylor series; C=Cornfield; M=Mid-P; F=Fisher Exact)			
STATISTICAL TESTS	Chi-square	1-tailed p	2-tailed p
Chi-square - uncorrected	7,7973		0,0052323437
Chi-square - Mantel-Haenszel	7,7634		0,0053314602
Chi-square - corrected (Yates)	6,8678		0,0087763081
Mid-p exact		0,0034497339	
Fisher exact 1-tailed		0,0050509149	0,0093874245
